# 518 3D Monitoring of Burn Wounds Treated with Microporous Annealing Particle Hydrogel in a Porcine Model

**DOI:** 10.1093/jbcr/iraf019.147

**Published:** 2025-04-01

**Authors:** Alekhya Gurram, Isabelle Bergman, Juquan Song, Kan Nakamoto, Julia Kleinhapl, Steven Wolf, Amina El Ayadi

**Affiliations:** University of Texas Medical Branch - John Sealy School of Medicine; University of Texas Medical Branch; University of Texas Medical Branch; University of Texas Medical Branch; University of Texas Medical Branch; University of Texas Medical Branch; University of Texas Medical Branch

## Abstract

**Introduction:**

Split-thickness skin grafts (STSG) are the standard of care in many burn centers. However, donor skin availability and the length/complexity of grafting surgeries may limit their use. Existing hydrogels and biomaterials can help with wound closure but have limited efficacy in tissue regeneration. The microporosity, injectability, and modular assembly inherent to the novel microporous annealed particle (MAP) scaffolds can facilitate cell migration, resulting in improved wound healing.

**Methods:**

Using the Red Duroc Pig model of burn excision and grafting, we assessed the efficacy of MAP hydrogel compared to a control hydrogel (polyethylene Glycol or PEG) and STSG alone. On days 28, 60, 90, and 120 after burn excision and treatment, a 3D camera was used to take images of burn wounds throughout the healing process. The 3D analysis software measured the following skin/scar parameters: wound perimeter, area, volume, height, depth, diameter, and roughness.

**Results:**

MAP hydrogel and STSG, in general, show significantly less wound contracture compared to PEG hydrogel. MAP Hydrogel has a significantly greater positive volume, defined as volume above the software-established reference plane, than STSG alone. STSG alone significantly increased negative volume below the software-established reference plane, compared to MAP hydrogel and PEG hydrogel. MAP-treated wounds show significantly higher diameter compared to STSG alone at Days 90 and 120 and compared to PEG hydrogel at Days 60 and 90. MAP and PEG hydrogels show significantly less roughness compared to autograft on Day 28, and MAP hydrogel and autograft show significantly greater roughness compared to PEG hydrogel.

**Conclusions:**

MAP hydrogel promotes wound healing by accelerating re-epithelialization and by reducing contraction and epidermal thickness towards the later stages of healing. MAP hydrogel shows promise as a treatment for burn wounds that can reduce donor skin use due to its ability to limit wound contracture, allowing for better tissue repair.

**Applicability of Research to Practice:**

This research offers practical value by informing physicians of a new therapeutic option that enhances healing and minimizes scarring in burn patients. This specific project is particularly beneficial as it employs 3D analysis to confirm these improvements in healing and scarring.

**Funding for the Study:**

N/A

